# Combined opioid-sodium aescinate therapy in blunt thoracic trauma: retrospective evaluation

**DOI:** 10.3389/fphar.2025.1623916

**Published:** 2025-09-19

**Authors:** Jun Zhang, Yijie Yan, Zhiyu Guan

**Affiliations:** Department of Thoracic Surgery, The Second Hospital of Tianjin Medical University, Tianjin, China

**Keywords:** blunt thoracic trauma, opioids, sodium aescinate, analgesic effect, combined therapy

## Abstract

**Background:**

Effective analgesia is crucial for patients with blunt thoracic trauma, yet the optimal analgesic approach remains controversial. This study aimed to evaluate the efficacy of opioids combined with Sodium Aescinate in blunt thoracic trauma management.

**Methods:**

Fifty patients with blunt thoracic trauma were randomly assigned to receive either opioids alone (morphine hydrochloride sustained-release tablets (MHST), Group A) or opioids combined with Sodium Aescinate (Group B). Pain scores, respiratory parameters, complications, and hospitalization metrics were assessed.

**Results:**

When pain number rating scale (NRS) scores reached ≤4, Group B required significantly lower opioid doses throughout therapy. Group B demonstrated significantly higher FEV1, FVC, and arterial PO2, and lower PCO2 compared to Group A, while respiratory rates remained similar between groups. Opioid-related complications (nausea, constipation) were significantly reduced in Group B, which also experienced shorter hospital stays and lower costs.

**Conclusion:**

This study demonstrated synergism between opioids and Sodium Aescinate in providing effective analgesia. The combination therapy offers an efficient and economical approach for pain management in blunt thoracic trauma, with improved respiratory function and reduced opioid-related complications.

## Introduction

Thoracic trauma represents one of the most significant causes of mortality and morbidity in trauma patients worldwide, with substantial clinical and socioeconomic implications. Blunt thoracic trauma carries an alarming mortality rate ranging from 10% to 25% (Hoogma et al., 2024). The management of blunt thoracic trauma has been subject to ongoing clinical debate and evolution over decades. Traditionally, treatment protocols have centered around two fundamental principles: immobilization and analgesia. However, research focus has been disproportionately directed toward various immobilization techniques, including mechanical stabilization methods and ventilatory support strategies (Gamberini et al., 2025; Gola et al., 2020). This research imbalance has resulted in relatively fewer evidence-based innovations in pain management protocols despite its critical importance. Current literature increasingly recognizes that pain control and immobilization should be considered equally essential components in the comprehensive management of thoracic trauma patients.

Effective pain control represents a crucial determinant of patient outcomes following thoracic trauma. The successful management of pain has been demonstrated to significantly reduce morbidity and enhance overall patient satisfaction during the recovery process (Feray et al., 2023). Pain associated with blunt thoracic trauma, particularly in cases involving rib fractures, can severely impair respiratory mechanics, leading to reduced lung volumes, inadequate clearance of secretions, and potentially progressive atelectasis (Cha et al., 2021; Chin et al., 2021). These complications can subsequently result in pneumonia, respiratory failure, and prolonged hospitalization if pain remains inadequately controlled. Despite this clinical significance, comprehensive research specifically examining pain control strategies in thoracic trauma remains relatively limited compared to other aspects of management (Koushik et al., 2023; Lazar et al., 2024; Lodhia et al., 2023; Mohamed et al., 2024; Mukherjee et al., 2023; Niazi et al., 2025). Sodium aescinate (SA), a triterpene saponin extract from horse chestnut seeds, demonstrates multiple therapeutic properties and finds extensive application in clinical practice for treating edema and inflammatory conditions (Xi et al., 2025). Studies have investigated sodium aescinate in acute lung injury. It can mitigate oleic acid-induced acute lung injury by modulating the levels of SOD, MDA, and MMP-9 in plasma and lung tissue (Wei et al., 2011). However, research on sodium aescinate in blunt thoracic trauma remains limited.

To explore novel approaches for pain management after trauma, we retrospectively analyzed patient data comparing the effects of a combination therapy of opioids and glycyrrhetinic acid (saponin) with monotherapy using opioids alone. Our analysis focused on three primary outcomes: assessing the differences in analgesic effects between the two groups, evaluating the impact of each treatment on respiratory parameters, and determining the effectiveness of the combined therapy in mitigating adverse effects associated with opioid use alone.

## Methods

### Study design and ethical considerations

This retrospective study consecutively screened inpatients diagnosed with blunt thoracic trauma at the Second Hospital of Tianjin Medical University between January 2017 and January 2023. We confirm that the parent trial has not been published, and all the data originates from the hospital medical record database. The protocol received ethical approval from the institutional review board of Tianjin Medical University (Approval No.KY 2025K294) and adhered to the Declaration of Helsinki principles. Written informed consent was obtained from all participants prior to data collection.

### Participant selection criteria

**FIGURE 1:**
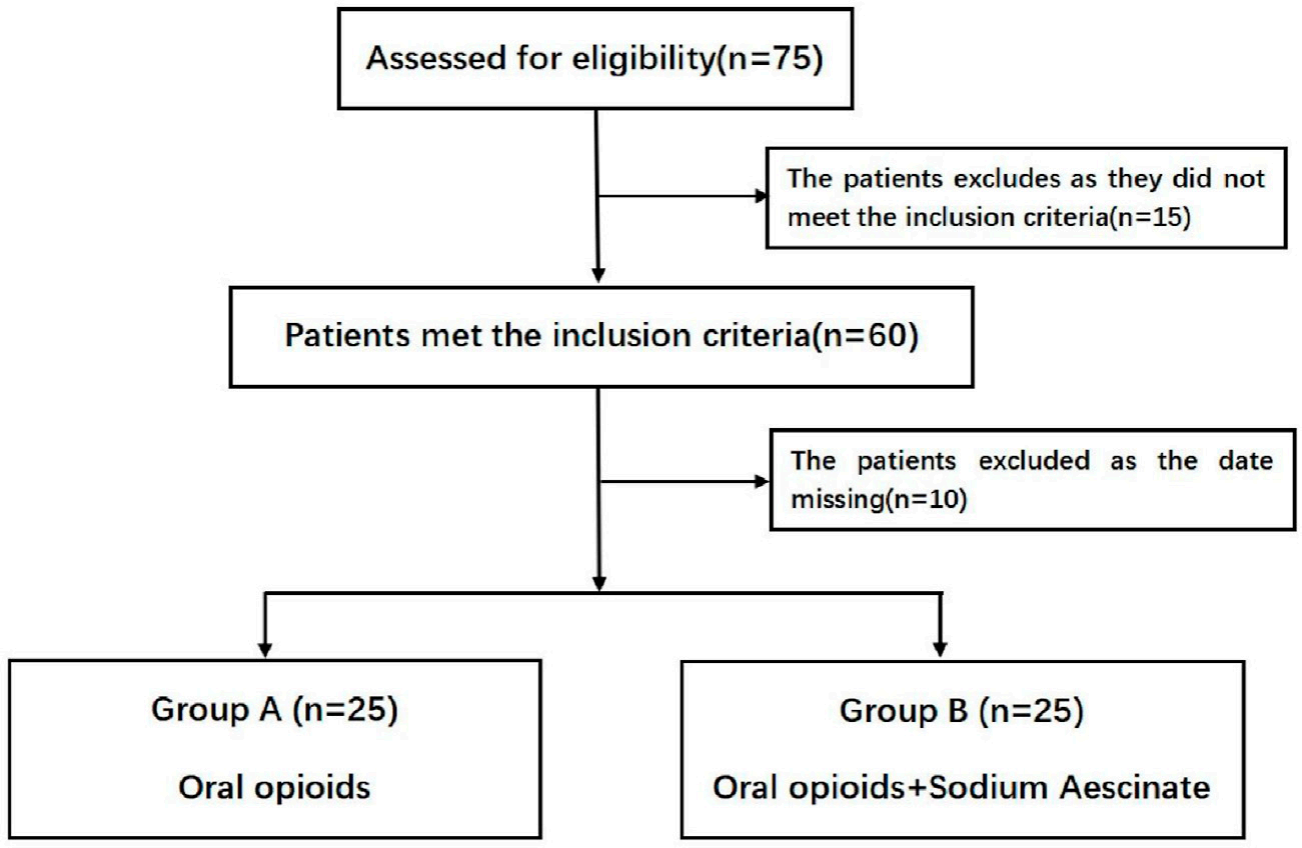
The entire study sample data inclusion process.

Inclusion required radiologically confirmed blunt thoracic trauma involving soft tissue injuries or bony fractures (rib fractures/flail chest). Exclusion criteria comprised: 1) polytrauma with Injury Severity Score (ISS) > 25; 2) chronic pain disorders or cancer-related pain syndromes; 3) severe cardiopulmonary/renal/hepatic insufficiency (Child-Pugh C or eGFR <30 mL/min/1.73 m^2^); 4) active malignancies; 5) body mass index (BMI) > 30 kg/m^2^; 6) tobacco use (>10 cigarettes/day) or alcohol abuse (>3 standard drinks/day); 7) incomplete clinical records. A total of 75 patients met eligibility criteria and completed follow-up (Figure 1).

### Clinical data collection

Demographic parameters (age, sex, BMI) and injury characteristics (ISS, fracture patterns) were systematically recorded (Table 1). Respiratory function was assessed through serial measurements of respiratory rate (RR), arterial blood gases (PaO_2_/PaCO_2_), and spirometry (FVC/FEV_1_). Pain intensity was quantified using the 11-point Numerical Rating Scale (NRS: 0 = no pain, 10 = worst imaginable pain) under standardized resting conditions.

**TABLE 1:** Comparison of baseline characteristics, complications, and hospitalization parameters between groups A and B.

Group	A (monotherapy)	B (combination therapy)	*P* value
Cases	25	25	
Gender (male:female)	16/9	18/7	0.07
Age (Year, mean ± sd)	52.6 ± 6.1	54.1 ± 4.0	0.423
ISS (mean ± sd)	13.1 ± 2.0	13.2 ± 1.7	0.962
BMI (mean ± sd)	23.3 ± 1.7	23.6 ± 1.4	0.395
NRS-0h			1
8	25 (100%)	25 (100%)	
NRS-0.5 h			0.8
7	14 (56%)	13 (52%)	
8	11 (44%)	12 (48%)	
NRS-2h			0.5
6	0 (0%)	2 (8.0%)	
7	20 (80%)	20 (80%)	
8	5 (20%)	3 (12%)	
NRS-4h			0.8
6	9 (36%)	10 (40%)	
7	16 (64%)	14 (56%)	
8	0 (0%)	1 (4.0%)	
NRS-8h			0.2
5	0 (0%)	4 (16%)	
6	18 (72%)	16 (64%)	
7	7 (28%)	5 (20%)	
NRS-24 h			<0.001
4	0 (0%)	1 (4.0%)	
5	5 (20%)	18 (72%)	
6	19 (76%)	6 (24%)	
7	1 (4.0%)	0 (0%)	
NRS-36 h			0.2
4	0 (0%)	3 (12%)	
5	19 (76%)	18 (72%)	
6	6 (24%)	4 (16%)	
NRS-48 h			0.2
4	8 (32%)	14 (56%)	
5	16 (64%)	11 (44%)	
6	1 (4.0%)	0 (0%)	
NRS-72 h			0.3
4	21 (84%)	24 (96%)	
5	4 (16%)	1 (4.0%)	
Nausea (n,%)	10 (40%)	3 (12%)	0.043
Vomiting (n,%)	3 (12%)	1 (4%)	0.602
Hypnosia (n,%)	1 (4%)	0 (0%)	1
Constipation (n,%)	9 (36%)	3 (12%)	0.047
Hospitalization expenses [d,M (QR)]	(38214.5,44505.0)40984.0	(20515.5,24051.5)23018.0	0.038
Length of stay [d,M (QR)]	(14.5,18.0)16.0	(9.0,11.5)10.0	0.021

Pain scores (NRS), respiratory function parameters (FVC, FEV_1_, PaO_2_, PaCO_2_), and cumulative opioid consumption were measured at baseline (pre-admission or pre-treatment) and at nine post-treatment timepoints (6, 12, 24, 36, 48, 60, 72, 84, and 96 h).

### Pharmacological intervention and outcome measures

Patients were randomly allocated to either the control group (A) receiving MHST (10 mg PO BID) or the experimental group (B) receiving combined therapy of MHST (10 mg PO BID) plus sodium aescinate tablets (30 mg PO BID). Rescue analgesia with 10 mg MHST was administered when NRS scores exceeded 4 points post-intervention, and medication adherence was monitored through pill counts and pharmacy records (the NRS ≥4 threshold aligns with International Association for the Study of Pain guidelines for moderate pain requiring intervention). Primary endpoints included pain control efficacy (defined as NRS ≤4) and opioid consumption (the unit is milligrams). Secondary outcomes encompassed hospitalization duration and costs, pulmonary function recovery (measured by FVC/FEV_1_ change from baseline), and analgesia-related complications (including nausea/vomiting incidence, somnolence duration, and constipation severity). Adverse events were documented using CTCAE v5.0 criteria through daily clinician assessments and patient self-reports.

Average daily opioid consumption (mg/day) was defined as the total cumulative opioid dose (mg) administered during the hospitalization divided by the length of hospital stay (days).

### Statistical analysis

Data analysis utilized SPSS 24.0 (IBM Corp.) with two-tailed α = 0.05. Normality was verified using Shapiro-Wilk tests (W > 0.90). Continuous variables were expressed as mean ± SD (parametric) or median [IQR] (non-parametric), analyzed via independent t-tests or Mann-Whitney U tests respectively. Categorical variables underwent χ^2^ or Fisher’s exact testing with effect sizes reported as odds ratios [95%CI]. Multivariable regression adjusted for age, ISS, and baseline NRS scores.

For the longitudinal data on respiratory function parameters and cumulative opioid consumption, a linear mixed-effects model was employed. Fixed effects included time (in hours), treatment group, and their interaction, while subjects were entered as random effects to account for individual variability. If the overall model was significant, *post hoc* comparisons at each time point were performed with Bonferroni correction.

## Results

### Participant enrollment and group allocation

A total of 75 patients with blunt thoracic trauma were initially identified and assessed for eligibility. Following the application of exclusion criteria, 25 patients were excluded from the study: 15 due to history of chronic pain and long-term analgesic use, and 10 due to incomplete data documentation. The remaining 50 patients were equally allocated into two treatment groups: Group A (n = 25) received standard (morphine hydrochloride sustained-release tablets) MHST alone, while Group B (n = 25) received MHST combined with Sodium Aescinate Tablets. Both groups demonstrated comparable baseline characteristics, allowing for meaningful between-group comparisons. All enrolled participants completed the study protocol with no dropouts recorded during the follow-up period.

### Analgesic requirements and opioid consumption patterns

**FIGURE 2:**
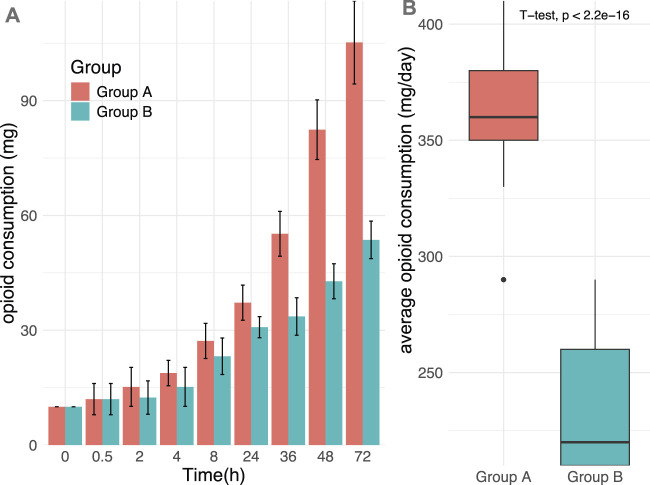
The time progression of opioid demand in two groups over time trajectories. **(A)** The trajectory of the change of opioid needs over time; **(B)** The trajectory of the change of opioid needs during the entire process. Group A = monotherapy. Group B = combination therapy.

Opioid consumption was systematically monitored over a 72-h period following trauma onset while maintaining pain scores at or below NRS ≤4 across both groups. Group B demonstrated significantly reduced MHST dosage requirements compared to Group A at multiple time points: at 24 h (30.8 ± 2.76 vs 37.2 ± 4.58), 36 h (33.6 ± 4.89 vs 55.2 ± 5.85), 48 h (42.8 ± 4.58 vs 82.4 ± 7.78), and 72 h (53.6 ± 4.89 vs 105.2 ± 10.84). This pattern resulted in a markedly lower cumulative opioid consumption in Group B compared to Group A (233.6 ± 28.71 vs 363.2 ± 27.04, p < 0.001). The temporal progression of opioid requirements exhibited distinct trajectories between groups, with Group A showing a steeper increase over time as illustrated in Figure 2.

### Respiratory function and physiological parameter improvements

**FIGURE 3:**
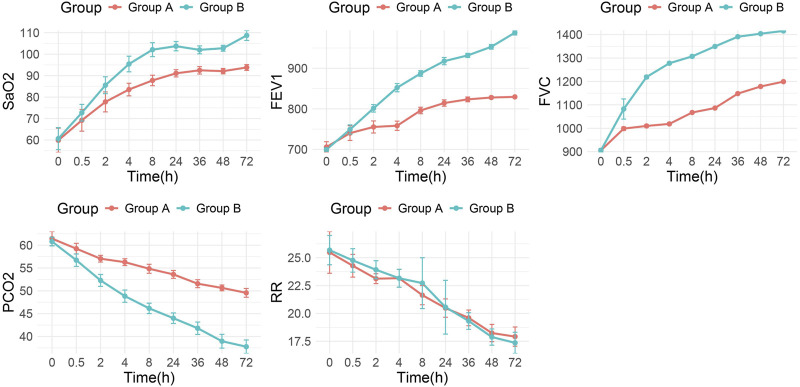
Trends of lung function parameters over time between the two groups. Group A = monotherapy. Group B = combination therapy.

Comprehensive assessment of physiological parameters revealed comparable baseline values between groups prior to intervention. Following treatment, both groups demonstrated significant improvements in pain scores, PO_2_, PCO_2_, respiratory rate (RR), forced vital capacity (FVC), and forced expiratory volume in one second (FEV_1_) compared to their respective baseline values (p < 0.001). Notably, Group B exhibited superior outcomes in several key parameters compared to Group A, including higher PO_2_ levels, greater improvements in FVC and FEV_1_, and lower PaCO_2_ values (p < 0.001 for all comparisons). Interestingly, no statistically significant difference was observed in respiratory rate between the two treatment groups (p > 0.05). The comparative improvements in pulmonary function parameters are graphically presented in Figure 3, demonstrating the enhanced respiratory recovery profile of patients receiving combination therapy.

### Healthcare utilization, safety profile, and economic outcomes

The safety assessment revealed no instances of severe respiratory depression or hypotension in either treatment group. However, significant differences emerged in healthcare utilization metrics and adverse event profiles. Patients in Group B exhibited a significantly shorter hospital length of stay and consequently lower hospitalization expenses compared to Group A (P < 0.05 for both measures). The incidence of opioid-associated complications, particularly nausea and constipation, was significantly reduced in Group B (P < 0.05), suggesting enhanced tolerability of the combination therapy. The rates of vomiting and somnolence were comparable between groups with no statistically significant differences observed. A comprehensive comparison of clinical outcomes, adverse events, and economic parameters is presented in Table 1, highlighting the multidimensional benefits of the combination therapy approach.

## Discussion

Our primary finding reveals that the combination of opioids and Sodium Aescinate effectively alleviates pain in blunt thoracic trauma patients while significantly reducing required opioid dosages. This reduction directly translated to a marked decrease in opioid-related adverse effects, including nausea and constipation. The combination therapy significantly improved respiratory parameters, with Group B (combination therapy) demonstrating higher FEV1 and FVC values and lower PCO2 levels compared to Group A (opioid monotherapy). These improved respiratory mechanics subsequently contributed to shorter hospital stays and reduced hospitalization costs for patients receiving the combined regimen. These findings are particularly relevant given that blunt thoracic trauma represents the majority of thoracic trauma cases and encompasses both soft tissue and bone injuries, including rib fractures that affect approximately two-thirds of chest trauma patients. The prevalence of complications in blunt thoracic trauma patients underscores the need for optimized treatments that minimize respiratory complications while providing adequate pain control. Future studies should validate whether age-specific benefits exist.

The efficacy of the combined therapy can be explained through complementary mechanisms of action. Pain in blunt chest trauma patients arises from multiple pathways, including inflammatory responses that lead to hyperemia, increased peripheral capillary permeability, and tissue exudation (Polycarpou and Kim, 2021; Schnadthorst et al., 2023). These processes result in tissue swelling and increased tension, exacerbating pain sensation. Sodium Aescinate appears to inhibit serum lysosomal activity, stabilize lysosomal membranes, prevent protease metabolism, and reduce capillary permeability—all contributing to decreased exudation and subsequent pain (Sharma et al., 2022; Stopenski et al., 2022). Additionally, it affects vascular endothelial cell receptors, causing constriction of capacitance vessels and enhancing venous return, which reduces venous congestion and tissue swelling. By addressing these underlying inflammatory mechanisms, Sodium Aescinate provides a pathway for pain relief that is complementary to the direct analgesic effects of opioids, thereby allowing for dose reduction of the latter while maintaining effective analgesia (Szamborski et al., 2022; van Zyl et al., 2024).

The management of blunt thoracic trauma has consistently posed significant challenges to thoracic surgeons. Despite extensive research, a universally recognized optimal treatment modality remains elusive. Current management strategies have centered on immobilization, chest physiotherapy, and effective pain control. Adequate analgesia remains the cornerstone of successful management as it enhances respiratory mechanics, whereas uncontrolled pain causes splinting and guarding, leading to reduced tidal volumes, decreased mobility, and impaired ability to cough and clear secretions (Sharma et al., 2022; Simon and Wickham, 2019). Various pain control strategies have been employed clinically, including intravenous narcotics, local rib blocks, pleural infusion catheters, paravertebral blocks, and epidural analgesia (Polycarpou and Kim, 2021). Each method presents distinct advantages and limitations. Epidural analgesia has gained advocacy in numerous studies due to its minimal effects on respiratory depression, somnolence, and gastrointestinal symptoms. However, it is not universally applicable due to several drawbacks. The procedure requires specialized technical expertise and carries potential complications including hypotension, epidural infections, hematoma, and spinal cord trauma (Almeida, 2021). It is contraindicated in patients with fever, coagulation abnormalities, or altered mental status, and some patients resist invasive procedures regardless of efficacy. Oral analgesics remain widely accepted due to their non-invasive nature, effectiveness, and ease of administration. While non-steroidal anti-inflammatory drugs can contribute to pain management in blunt thoracic trauma, they often provide insufficient relief, necessitating opioid supplementation (Cha et al., 2021; Chin et al., 2021; Ja et al., 2020; Kourouche et al., 2018).

However, the dose-dependent side effects of opioids (nausea, vomiting, drowsiness, and constipation) limit their widespread use. The study shows that traumatic injury significantly predisposes patients to chronic opioid use, and those who develop long-term consumption face a higher subsequent mortality risk (von Oelreich et al., 2020). Prolonged opioid exposure initiates regulatory adaptations of opioid receptors—such as desensitization and internalization—that culminate in receptor tolerance. These adaptations recruit several intracellular pathways, most prominently the upregulation of the cyclic AMP/PKA cascade and activation of cAMP response element–binding signaling (Wang et al., 2019). In a randomized study of outpatient laparoscopic cholecystectomy and appendectomy patients, a 3-day multimodal regimen of acetaminophen, ibuprofen, and up to five 5 mg rescue oxycodone tablets provided effective analgesia—patients used an average of only 1.8 oxycodone tablets, and 50% required no opioids at all (Sim et al., 2019). A meta-analysis showed that postoperative bleeding rates were comparable between ketorolac (33/1304, 2.5%) and control groups (21/1010, 2.1%; OR = 1.1, 95% CI 0.61–2.06, P = 0.72) (Wick et al., 2017). These studies demonstrate the need to reduce opioid use in clinical settings to lower patient mortality and addiction risks, making multimodal treatment strategies essential.

Our findings suggest several important clinical implications. First, by reducing opioid requirements, the combination therapy significantly diminishes respiratory inhibition that could otherwise impede expectoration and potentially lead to atelectasis and pulmonary infection. The absence of RR changes in Group B, despite improved PO_2_and FEV_1_, suggests enhanced alveolar ventilation efficiency and reduced dead space ventilation rather than altered respiratory drive. This may result from reduced Splinting Respiration due to better analgesia, permitting deeper tidal volumes without increasing respiratory frequency. This respiratory advantage may partially explain the improved clinical outcomes observed in the combination therapy group, including enhanced ventilation function, prevention of pulmonary complications, shortened hospital stays, and reduced hospitalization costs. Second, the combination therapy represents a non-invasive, safe, and easily administered alternative. This characteristic makes it particularly valuable for patients with contraindications to invasive procedures or those with preferences for non-invasive treatment options.

Despite these promising results, several limitations warrant consideration. Although no renal adverse events occurred in our cohort, sodium aescinate carries theoretical nephrotoxicity risks in vulnerable populations (e.g., pre-existing renal impairment). We recommend cautious use in patients with eGFR <60 mL/min/1.73 m^2^ and routine renal function monitoring during therapy. Our study did not stratify outcomes by age groups or injury severity, which could provide more nuanced insights into which patient populations might benefit most from this combination therapy. Additionally, long-term follow-up data would be valuable to assess any potential differences in chronic pain development or functional outcomes between treatment approaches. Future research should explore optimal dosing regimens, potential interactions with other commonly prescribed medications in trauma patients, and direct comparison with regional anesthetic techniques like paravertebral blocks or serratus plane blocks. Prospective multicenter trials with larger patient cohorts would strengthen the evidence base for this promising treatment approach.

## Conclusion

Effective analgesia remains crucial in the treatment of blunt thoracic trauma. The combination of opioids with Sodium Aescinate represents a promising approach that reduces opioid dosage requirements while enhancing patient ventilation function, preventing pulmonary complications, shortening hospital stays, and lowering treatment costs. Compared to other analgesic approaches, this oral medication regimen offers advantages of being non-invasive, safe, effective, and easily administered. These findings suggest that the combined use of opioids and Sodium Aescinate could significantly improve recovery outcomes following blunt thoracic trauma and merits consideration as a standard treatment option in appropriate clinical scenarios.

## Data Availability

The original contributions presented in the study are included in the article/Supplementary Material, further inquiries can be directed to the corresponding author.

## References

[B1] Almeida C. R. (2021). Parascapular sub-Iliocostalis plane block: comparative Description of a novel technique for Posterior rib fractures. Pain Pract. 21 (6), 708–714. 10.1111/papr.13003 33586285

[B2] Cha P. I. Min J. G. Patil A. Choi J. Kothary N. N. Forrester J. D. (2021). Efficacy of intercostal cryoneurolysis as an analgesic adjunct for chest wall pain after surgery or trauma: systematic review. Trauma Surg. Acute Care Open 6 (1), e000690. 10.1136/tsaco-2021-000690 34079913 PMC8137159

[B3] Chin K. J. Versyck B. Pawa A. (2021). Ultrasound-guided fascial plane blocks of the chest wall: a state-of-the-art review. Anaesthesia 76 (Suppl. 1), 110–126. 10.1111/anae.15276 33426660

[B4] Feray S. Lemoine A. Aveline C. Quesnel C. (2023). Pain management after thoracic surgery or chest trauma. Minerva Anestesiol. 89 (11), 1022–1033. 10.23736/S0375-9393.23.17291-9 37671536

[B5] Gamberini L. Moro F. Dallari C. Tartaglione M. Mazzoli C. A. Allegri D. (2025). Regional anesthesia modalities in blunt thoracic trauma: a systematic review and Bayesian network meta-analysis. Am. J. Emerg. Med. 89, 199–208. 10.1016/j.ajem.2024.12.029 39740311

[B6] Gola W. Zajac M. Cugowski A. (2020). Adjuvants in peripheral nerve blocks - the current state of knowledge. Anaesthesiol. Intensive Ther. 52 (4), 323–329. 10.5114/ait.2020.98213 33165883 PMC10183787

[B7] Hoogma D. F. Brullot L. Coppens S. (2024). Get your 7-point golden medal for pain management in video-assisted thoracoscopic surgery. Curr. Opin. Anaesthesiol. 37 (1), 64–68. 10.1097/ACO.0000000000001325 38085865

[B8] Jack J. M. McLellan E. Versyck B. Englesakis M. F. Chin K. J. (2020). The role of serratus anterior plane and pectoral nerves blocks in cardiac surgery, thoracic surgery and trauma: a qualitative systematic review. Anaesthesia 75 (10), 1372–1385. 10.1111/anae.15000 32062870

[B9] Kourouche S. Buckley T. Munroe B. Curtis K. (2018). Development of a blunt chest injury care bundle: an integrative review. Injury 49 (6), 1008–1023. 10.1016/j.injury.2018.03.037 29655592

[B10] Koushik S. S. Bui A. Slinchenkova K. Badwal A. Lee C. Noss B. O. (2023). Analgesic techniques for rib fractures-A comprehensive review article. Curr. Pain Headache Rep. 27 (11), 747–755. 10.1007/s11916-023-01172-9 37747621

[B11] Lazar A. E. Butiulca M. Farczadi L. (2024). Challenges of the regional anesthetic techniques in intensive care Units - a narrative review. J. Crit. Care Med. Targu Mures. 10 (3), 198–208. 10.2478/jccm-2024-0023 39108409 PMC11295270

[B12] Lodhia J. V. Eyre L. Smith M. Toth L. Troxler M. Milton R. S. (2023). Management of thoracic trauma. Anaesthesia 78 (2), 225–235. 10.1111/anae.15934 36572548

[B13] Mohamed E. H. Elmoheen A. Bashir K. Fayed M. Abdurabu M. Abdelrahim M. G. (2024). Comparative analysis of intravenous opioids versus thoracic epidural anesthesia in fractured rib pain management: a systematic review and meta-analysis. Cureus 16 (1), e51740. 10.7759/cureus.51740 38318591 PMC10840374

[B14] Mukherjee K. Schubl S. D. Tominaga G. Cantrell S. Kim B. Haines K. L. (2023). Non-surgical management and analgesia strategies for older adults with multiple rib fractures: a systematic review, meta-analysis, and joint practice management guideline from the Eastern Association for the Surgery of Trauma and the Chest Wall Injury Society. J. Trauma Acute Care Surg. 94 (3), 398–407. 10.1097/TA.0000000000003830 36730672

[B15] Niazi A. U. Solish M. Moorthy A. Niazi F. Abate A. H. Devion C. (2025). Use of fascial plane blocks for traumatic rib fractures: a scoping review. Reg. Anesth. Pain Med.–2024-106366. 10.1136/rapm-2024-106366 40107733

[B16] Polycarpou A. Kim B. D. (2021). Pediatric surgical rib fixation: a collected case series of a rare entity. J. Trauma Acute Care Surg. 91 (6), 947–950. 10.1097/TA.0000000000003376 34407006

[B17] Schnadthorst P. G. Lankes C. Schulze C. (2023). Treatment of trauma-related vertebral body fractures of the thoracic and lumbar spine with orthotic devices: a review. Unfallchirurgie (Heidelb) 126 (8), 632–642. 10.1007/s00113-022-01195-8 35849146

[B18] Sharma R. Louie A. Thai C. P. Dizdarevic A. (2022). Chest wall nerve blocks for Cardiothoracic, breast surgery, and rib-related pain. Curr. Pain Headache Rep. 26 (1), 43–56. 10.1007/s11916-022-01001-5 35089532

[B19] Sim V. Hawkins S. Gave A. A. Bulanov A. Elabbasy F. Khoury L. (2019). How low can you go: achieving postoperative outpatient pain control without opioids. J. Trauma Acute Care Surg. 87 (1), 100–103. 10.1097/TA.0000000000002295 31259870

[B20] Simon J. B. Wickham A. J. (2019). Blunt chest wall trauma: an overview. Br. J. Hosp. Med. (Lond). 80 (12), 711–715. 10.12968/hmed.2019.80.12.711 31822181

[B21] Stopenski S. Binkley J. Schubl S. D. Bauman Z. M. (2022). Rib fracture management: a review of surgical stabilization, regional analgesia, and intercostal nerve cryoablation. Surg. Pract. Sci. 10, 100089. 10.1016/j.sipas.2022.100089 39845586 PMC11750013

[B22] Szamborski M. Janc J. Rosinczuk J. Janc J. J. Lesnik P. Lysenko L. (2022). Use of ultrasound-guided interfascial plane blocks in anterior and lateral thoracic wall region as safe method for patient anesthesia and analgesia: review of techniques and approaches during COVID-19 pandemic. Int. J. Environ. Res. Public Health 19 (14), 8696. 10.3390/ijerph19148696 35886547 PMC9320164

[B23] van Zyl T. Ho A. M. Klar G. Haley C. Ho A. K. Vasily S. (2024). Analgesia for rib fractures: a narrative review. Can. J. Anaesth. 71 (4), 535–547. 10.1007/s12630-024-02725-1 38459368

[B24] von Oelreich E. Eriksson M. Brattstrom O. Sjolund K. F. Discacciati A. Larsson E. (2020). Risk factors and outcomes of chronic opioid use following trauma. Br. J. Surg. 107 (4), 413–421. 10.1002/bjs.11507 32031251

[B25] Wang S. C. Chen Y. C. Lee C. H. Cheng C. M. (2019). Opioid addiction, genetic susceptibility, and medical treatments: a review. Int. J. Mol. Sci. 20 (17), 4294. 10.3390/ijms20174294 31480739 PMC6747085

[B26] Wei T. Tong W. Wen-ping S. Xiao-hui D. Qiang X. Tian-shui L. (2011). The impact of sodium aescinate on acute lung injury induced by oleic acid in rats. Exp. Lung Res. 37 (10), 585–599. 10.3109/01902148.2011.622426 22087513

[B27] Wick E. C. Grant M. C. Wu C. L. (2017). Postoperative multimodal analgesia pain management with nonopioid analgesics and techniques: a review. JAMA Surg. 152 (7), 691–697. 10.1001/jamasurg.2017.0898 28564673

[B28] Xi C. Zhou J. Zheng X. Fu X. Xie M. (2025). Sodium aescinate-induced hepatotoxicity via ATF4/GSH/GPX4 axis-mediated ferroptosis. Sci. Rep. 15 (1), 1141. 10.1038/s41598-024-79723-2 39774712 PMC11706965

